# Effects of Nifedipine Tablets Combined With Magnesium Sulfate on Blood Coagulation Index, Oxidative Stress, NO and ET-1 Levels in Patients With Pregnancy Hypertension

**DOI:** 10.3389/fsurg.2022.862676

**Published:** 2022-03-28

**Authors:** Xiaomei Yu, Qiang Zhou

**Affiliations:** Department of Obstetrics, Weifang People's Hospital, Weifang, China

**Keywords:** nifedipine tablets, magnesium sulfate, pregnancy-induced hypertension, coagulation indicators, oxidative stress, NO, ET-1

## Abstract

**Objective:**

To explore the effects of nifedipine tablets combined with magnesium sulfate on blood coagulation indexes, oxidative stress and levels of NO and ET-1 in patients with Pregnancy-induced hypertension syndrome (PIH).

**Methods:**

A total of 110 patients with hypertension during pregnancy were admitted to our hospital from January 2020 to January 2021. According to the random number table method, 110 patients were divided into the control group and the therapy group, with 55 cases in each group. The blood pressure levels (systolic and diastolic blood pressure), coagulation indexes (TT, PT, APTT, Fib), oxidative stress indexes (LPO, MDA, SOD), vascular endothelial function (ET-1, NO), clinical efficacy and adverse reactions of the two groups were compared.

**Results:**

After therapy, the systolic blood pressure and diastolic blood pressure of the two groups were significantly decreased, and the therapy group was significantly lower than the control group (*P* < 0.05). After therapy, PT, TT, and APTT in two groups were significantly increased, and Fib was significantly decreased, and PT, TT, APTT in the therapy group were higher than those in the control group, and Fib was lower than that in the control group (*P* < 0.05). After therapy, LPO and MDA in two groups were significantly decreased, and SOD was significantly increased, and LPO and MDA in the therapy group were lower than those in the control group, and SOD was higher than that in the control group (*P* < 0.05). After therapy, ET-1 in two groups were significantly increased, and NO and ET-1/NO was significantly decreased, and ET-1 in the therapy group was higher than that in the control group, and NO and ET-1/NO were lower those in the control group (*P* < 0.05). The total clinical effective rate of patients in the therapy group was 94.5%, and in the control group was 81.8%, the therapy group was significantly better than the control group (*P* < 0.05). The total incidence of adverse reactions in the therapy group was 7.3%, and in the control group was 21.8%, the therapy group was significantly lower than the control group (*P* < 0.05).

**Conclusion:**

Nifedipine tablets combined with magnesium sulfate in the treatment of PIH can improve the blood coagulation function of patients, reduce oxidative stress damage, adjust the serum levels of ET-1 and NO, and improve the clinical efficacy.

## Introduction

Pregnancy-induced hypertension syndrome (PIH) is also known as pregnancy-induced hypertension. It usually occurs at 20 weeks of gestation or 2 weeks after delivery. In addition to high blood pressure, it can also be accompanied by edema, proteinuria, thrombocytopenia, and liver Clinical manifestations such as functional impairment (1, 2). PIH is more harmful to mothers and babies. It can cause high-risk complications such as miscarriage and postpartum hemorrhage. It greatly increases the risk of maternal delivery and affects the outcome of pregnancy. It is one of the main factors leading to maternal and perinatal death. Studies have shown that PIH is not treated in time, it will cause systemic dysfunction, secondary coma, convulsions, etc., and the main lesion of PIH is the spasm of arterioles in the whole body, which leads to the vasospasm of important organs in the whole body. Therefore, it is necessary not only to effectively control blood pressure during treatment, the key is to relieve the spasm (3). At present, the clinical treatment of hypertension in pregnancy is mainly based on drug therapy, which to a certain extent can help relieve patients' spasms, reduce patients' blood pressure, and reduce and improve cardiac load at the same time. However, in the course of drug treatment, certain drugs will inevitably cause a certain degree of damage to the fetus. Therefore, care must be taken in the choice of drugs during the treatment process. At present, the main drugs used in the treatment of pregnancy-induced hypertension are magnesium sulfate and nifedipine tablets. Nifedipine tablets can inhibit the influx of calcium ions into the cells by obstructing the membrane transport of calcium ions in the myocardium and vascular smooth muscle, which increases the coronary blood flow and improves the tolerance of the myocardium to ischemia, thereby achieving the effect of lowering blood pressure (4, 5). Magnesium sulfate can inhibit the central nervous system, relax skeletal muscles, have the effects of sedation, antispasm, and reduce intracranial pressure. It is often used to treat convulsions, eclampsia, uremia, tetanus and hypertensive encephalopathy (6). Nitric oxide (NO) is the most representative endogenous relaxing factor, and Endothelin-1 (ET-1) is a strong vasoconstrictor found so far, which is related to inflammatory reactions In the process, monocyte infiltration is related, and it is a type of inflammatory cytokines with chemotactic function (7, 8), but the expression of the two in the serum of gestational diabetes patients is currently unclear. Therefore, the purpose of this study is to treat patients with pregnancy-induced hypertension through the clinical efficacy of nifedipine tablets combined with magnesium sulfate intervention, as well as the effects of patients' blood coagulation, oxidative stress indicators, and NO and ET-1 levels.

## Materials and Methods

### General Information and Grouping

A total of 110 patients with hypertension during pregnancy were admitted to our hospital from January 2020 to January 2021. According to the random number table method, 110 patients were divided into the control group and the therapy group, with 55 cases in each group. The patients in the therapy group were 21–36 years old, with an average age of (29.64 ± 2.13) years; 40 cases of primiparas and 15 cases of postpartum women; gestational age was 32–40 weeks, with an average of (36.02 ± 2.13) weeks; systolic blood pressure was 141–165 mmHg, average systolic blood pressure (153.64 ± 2.13) mmHg; diastolic blood pressure 93–109 mmHg, average diastolic blood pressure (96.15 ± 2.58) mmHg. The control group was 22–35 years old, with an average age of (28.95 ± 2.06) years; 38 cases of primiparous women and 17 cases of postpartum women; gestational age was 33–39 weeks, with an average of (35.67 ± 2.01) weeks; systolic blood pressure was 140–161 mmHg, The average systolic blood pressure was (151.38 ± 2.06) mmHg; the diastolic blood pressure was 91–105 mmHg, and the average diastolic blood pressure was (93.06 ± 2.11) mmHg. All patients met the relevant diagnostic criteria of our hospital for pregnancy-induced hypertension. There was no statistically significant difference between the two groups of patients in general information such as age, gestational age, and birth experience (*P* > 0.05).

### Inclusion Criteria

All patients were diagnosed with pregnancy-induced hypertension, systolic blood pressure ≧140 mmHg and diastolic blood pressure ≧90 mmHg, accompanied by proteinuria 0.3 g/24 h, or random urine protein (+). All patients underwent routine fundus examination, blood coagulation function examination, biochemical index examinations such as heart, liver, kidney function, blood electrolytes, fetal growth and development index examination, cardiac ultrasonography, liver, gallbladder, spleen and kidney ultrasound examination to rule out important organ diseases. This subject was approved by the hospital ethics committee, and the patients and their families voluntarily participated in this experiment and signed an informed consent form.

### Exclusion Criteria

Patients with history of hypertension, history of diabetes, abnormal blood biochemistry and urine tests before pregnancy. Patients with liver, kidney and other organ diseases. Those who were allergic to the drugs used in this study. Complicated with hematological diseases. Combined with malignant tumor. Cognitive impairment. Incomplete clinical data.

### Treatment Methods

Patients in the control group were given magnesium sulfate intervention treatment. The total dosage of magnesium sulfate was 20 g for 24 h on the 1st day; the loading dose was 25% magnesium sulfate injection (Hangzhou Minsheng Medical Liquid Co., Ltd., product batch number 1409281, National Medicine Standard H33021961) 20 ml (5 g) Dilute with 20 ml of 5% glucose injection, and slowly inject intravenously within 15–20 min; then used a maintenance meter, that was, 60 ml (15 g) of 25% magnesium sulfate and dilute with 500 ml of 5% glucose injection for intravenous infusion, 1–2 g/h intravenous drip for maintenance. After that, 60 ml (5 g) of magnesium sulfate was used daily. Knee tendon reflex examinations should be done regularly before and during medication to measure the patient's breathing frequency and urine output. If the knee tendon reflex was found to be significantly weakened or disappeared, or the number of breaths was <16 times/min, the patient's urine output was <25 ml/h, the medication should be stopped immediately, 7 days as a course of treatment. On the basis of the control group, the therapy group was given nifedipine (CSPC Ouyi Pharmaceutical Co., Ltd., approval number: H13021315), 3 times a day, 10 mg each time, orally with warm water, 7 days as a course of treatment.

The principle of medication is to be treated in strict accordance with the treatment standards for hypertension in pregnancy in the Guidelines for the Diagnosis and Treatment of Hypertension in Pregnancy. During the administration and treatment of the two groups of patients, the relevant medical staff should pay close attention to the patients' usual urine output, fetal heart rate, respiration, and magnesium ion levels to prevent the patients from experiencing poisoning symptoms. And during the treatment, the patient's blood pressure, heart rate, and breathing changes are recorded in detail, and adverse reactions and changes in symptoms such as dizziness and headache are observed. Record the occurrence of pregnancy complications.

### Observation Indicators

#### Blood Pressure Detection

The systolic blood pressure (SBP) and diastolic blood pressure (DBP) of the two groups of patients before and after therapy were measured and compared.

#### Coagulation Index

Before and after therapy, 5 ml of fasting cubital venous blood was collected from the two groups of patients in the morning during the obstetric examination, centrifuged at 3,500 r/min for 10 min to take the upper serum, and placed in the refrigerator at −80°C for later use. Use CA8000 automatic blood coagulation analyzer (Syames, Japan) and supporting kits to measure coagulation function related indexes, including prothrombin time (PT), thrombin time (TT), fibrinogen (Fib), activated thrombin Original time (APTT).

#### Oxidative Stress Indicators

Take the sera of the two groups of patients in 1.5.2, and ELISA kit (Jiangsu Kejing Biological Company) to detect the serum levels of lipid peroxide (LPO), malondialdehyde (MDA), and superoxide dismutase (SOD).

#### Vascular Endothelial Function

Take the serum of the two groups of patients in 1.5.2, use the BS-800 automatic biochemical analyzer (Zhengzhou Nanbei Instrument Equipment Co.Ltd.) to detect serum endothelin 1 (ET-1), nitric oxide (NO) and ET-1/ NO level.

#### Judgment of Clinical Efficacy

Compare the clinical effects of the therapy group and the control group. Significantly effective: after therapy, the patient's systolic blood pressure is less than or equal to 140 mmHg, and the diastolic blood pressure is less than or equal to 90 mmHg; or the patient's systolic blood pressure drops >30 mmHg, and the diastolic blood pressure drops >15 mmHg; the patient's dizziness, headache and abdominal discomfort and other clinical symptoms have basically disappeared, and no postpartum complications appear. Effective: after therapy, the patient's systolic blood pressure is ≧140 and ≦150 mmHg, and the diastolic blood pressure is ≧90 and ≦100 mmHg. The clinical symptoms such as dizziness, headache, and abdominal discomfort are significantly improved, and no postpartum complications occur. Ineffective: after therapy, the patient has systolic blood pressure ≧150 mmHg, diastolic blood pressure ≧100 mmHg, clinical symptoms are not improved or even worsened, with mild edema and proteinuria.

#### Adverse Reactions

Observe and record the occurrence of adverse pregnancy outcomes such as premature birth, postpartum hemorrhage, fetal distress, and very low birth weight infants in the two groups.

### Statistical Methods

Graphpad priam 8.0 software was used for data analysis, and the measurement data were expressed as mean ± standard deviation. The *t*-test was in accordance with the normal distribution, and the Wilcoxon test was not in accordance with the normal distribution. The measurement data used χ^2^, and *P* < 0.05 indicated that the difference was statistically significant.

## Results

### Comparison of Blood Pressure Levels Between the Two Groups

**Figure 1:**
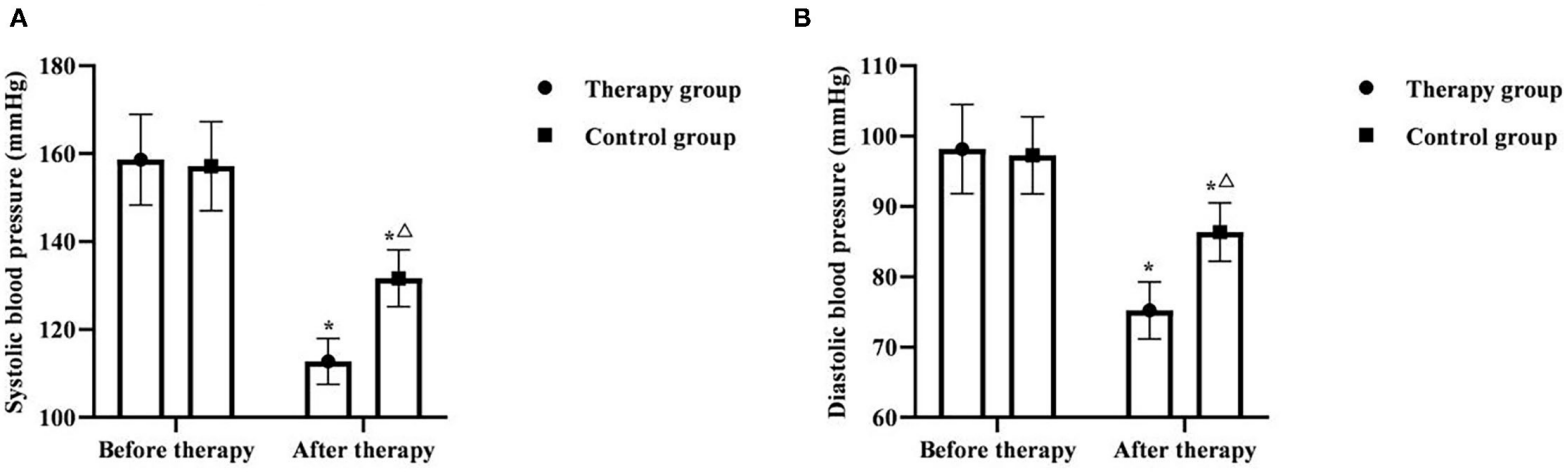
Comparison of blood pressure levels between the two groups (*x* ± *s, n* = 55). **(A)** was comparison of systolic blood pressure between the two groups. **(B)** was comparison of diastolic blood pressure between the two groups. *P < 0.05 was compared with the same group before therapy, ^Δ^*P* < 0.05 was compared with the control group after therapy.

Before therapy, there was no significant difference in systolic blood pressure and diastolic blood pressure between the two groups (*P* > 0.05). After therapy, the systolic blood pressure and diastolic blood pressure of the two groups were significantly decreased, and the therapy group was significantly lower than the control group (*P* < 0.05) (Figure 1).

### Comparison of Coagulation Indexes Between the Two Groups

**Figure 2:**
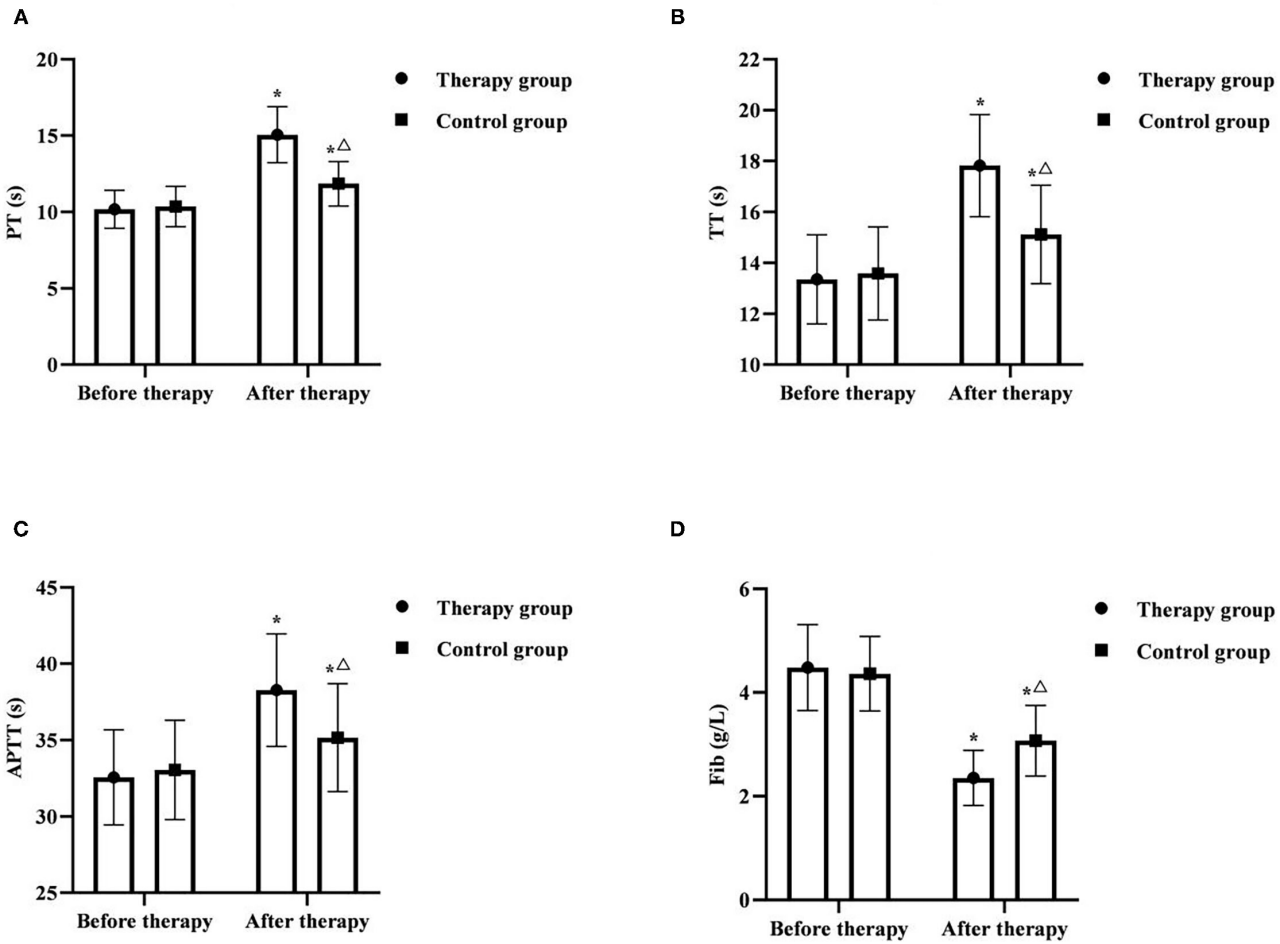
Comparison of coagulation indexes between the two groups (*x* ± *s, n* = 55). **(A)** was comparison of PT between the two groups. **(B)** was comparison of TT between the two groups. **(C)** was comparison of APTT between the two groups. **(D)** was comparison of Fib between the two groups. *P < 0.05 was compared with the same group before therapy, ^Δ^*P* < 0.05 was compared with the control group after therapy.

Before therapy, there was no significant difference in PT, TT, APTT, and Fib between the two groups (*P* > 0.05). After therapy, PT, TT, and APTT in two groups were significantly increased, and Fib was significantly decreased, and PT, TT, APTT in the therapy group were higher than those in the control group, and Fib was lower than that in the control group (*P* < 0.05) (Figure 2).

### Comparison of Oxidative Stress Indicators Between the Two Groups

**Figure 3:**
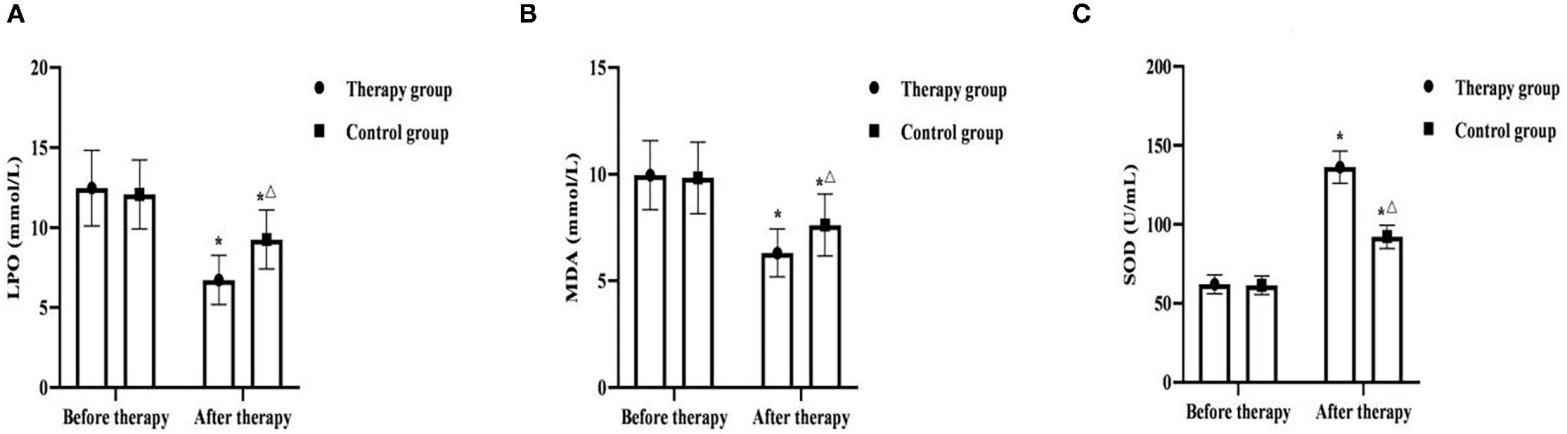
Comparison of oxidative stress indicators between the two groups (*x* ± *s, n* = 55). **(A)** was comparison of LPO between the two groups. **(B)** was comparison of MDA between the two groups. **(C)** was comparison of SOD between the two groups. *P < 0.05 was compared with the same group before therapy, ^Δ^*P* < 0.05 was compared with the control group after therapy.

Before therapy, there was no significant difference in LPO, MDA and SOD between the two groups (*P* > 0.05). After therapy, LPO and MDA in two groups were significantly decreased, and SOD was significantly increased, and LPO and MDA in the therapy group were lower than those in the control group, and SOD was higher than that in the control group (*P* < 0.05) (Figure 3).

### Comparison of Vascular Endothelial Function Between the Two Groups

**Figure 4:**
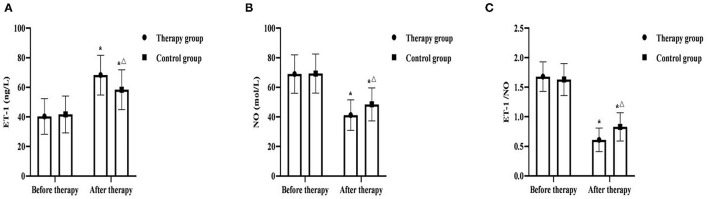
Comparison of vascular endothelial function between the two groups (*x* ± *s, n* = 55). **(A)** was comparison of ET-1 between the two groups. **(B)** was comparison of NO between the two groups. **(C)** was comparison of ET-1/NO between the two groups. *P < 0.05 was compared with the same group before therapy, ^Δ^*P* < 0.05 was compared with the control group after therapy.

Before therapy, there was no significant difference in ET-1, NO, ET-1/NO between the two groups (*P* > 0.05). After therapy, ET-1 in two groups were significantly increased, and NO and ET-1/NO was significantly decreased, and ET-1 in the therapy group was higher than that in the control group, and NO and ET-1/NO were lower those in the control group (*P* < 0.05) (Figure 4).

### Comparison of Clinical Efficacy Between the Two Groups

**Figure 5:**
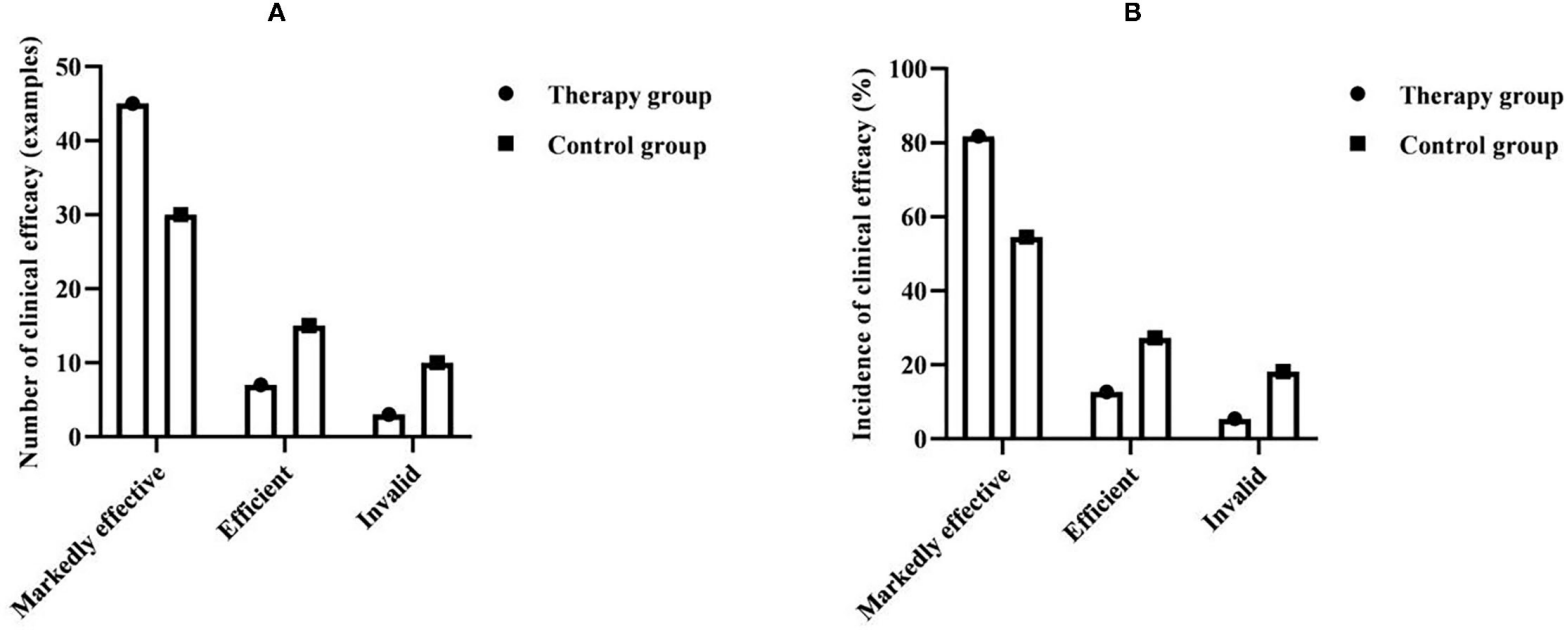
Comparison of clinical efficacy between the two groups (*n* = 55, %). **(A)** Comparison of the number of clinical efficacy between the two groups. **(B)** Comparison of the incidence of clinical efficacy between the two groups.

The total clinical effective rate of patients in the therapy group was 94.5%, and the total clinical effective rate of patients in the control group was 81.8%. Compared with the clinical efficacy between the groups, the therapy group was significantly better than the control group, and the difference was statistically significant (*P* < 0.05) (Table 1 and Figure 5).

**Table 1:** Comparison of clinical efficacy between the two groups (*n* = 55, %).

**Group**	**Markedly effective**	**Efficient**	**Invalid**	**Total effective**
Therapy group	45 (81.82)	7 (12.73)	3 (5.45)	52 (94.55)
Control group	30 (54.55)	15 (27.27)	10 (18.18)	45 (81.82)
*χ^2^*				4.274
*P*				0.039

### Comparison of Adverse Reactions Between the Two Groups

**Figure 6:**
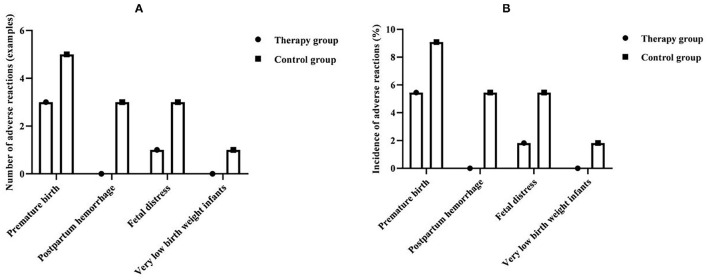
Comparison of adverse reactions between the two groups. **(A)** Comparison of the number of adverse reactions between the two groups. **(B)** Comparison of the incidence of adverse reactions between the two groups.

The total incidence of adverse reactions in the therapy group was 7.3%, and the total incidence of adverse reactions in the control group was 21.8%. The incidence of adverse reactions between the groups was significantly lower in the therapy group than in the control group, and the difference was statistically significant (*P* < 0.05) (Table 2 and Figure 6).

**Table 2:** Comparison of adverse reactions between the two groups (*n* = 55, %).

**Group**	**Premature birth**	**Postpartum hemorrhage**	**Fetal distress**	**Very low birth weight infant**	**Total incidence**
Therapy group	3 (5.45)	0 (0.00)	1 (1.82)	0 (0.00)	4 (7.27)
Control group	5 (9.09)	3 (5.45)	3 (5.45)	1 (1.82)	12 (21.82)
*χ^2^*					4.681
*P*					0.031

## Discussion

Pregnancy hypertension refers to a state in which both increased blood pressure and pregnancy coexist. It has a serious impact on the health of pregnant women and fetuses. If they cannot be effectively controlled in time, they will cause serious consequences with maternal or perinatal death (9). Therefore, how to effectively treat pregnancy-induced hypertension has always been the focus of clinical research. At present, drug therapy is the main treatment method used in clinical practice. The purpose of treatment is mainly to relieve spasm, expand blood volume, reduce blood pressure, sedation, diuresis and termination of pregnancy. However, in the course of drug treatment, certain drugs will inevitably cause a certain degree of harm to the fetus. Therefore, it is necessary to be cautious in the choice of drugs during treatment. Magnesium sulfate is the drug of choice for the treatment of hypertension in pregnancy. It can inhibit the central nervous system and block the conduction at the peripheral neuromuscular joints. It can calm, relieve spasm and relax skeletal muscles; it can also reduce intracranial pressure and relax peripherals. Vascular smooth muscle destroys sympathetic ganglion impulse conduction, promotes vasodilation and lowers blood pressure; in addition, magnesium sulfate also has the effect of accelerating protein metabolism, anti-inflammatory and de-seeding, thereby effectively alleviating the symptoms of proteinuria and edema in patients (10). However, the use of magnesium sulfate alone is prone to adverse reactions, and its use has certain limitations. It has been reported that the combined use of magnesium sulfate and nifedipine tablets can improve the effective rate of clinical treatment. Nifedipine is a calcium antagonist, which can relax vascular smooth muscle, expand blood vessels, reduce peripheral vascular resistance, lower blood pressure, and it can reduce afterload, suitable for long-term use, and can make up for the defects of magnesium sulfate (11). The results of this study showed that the blood pressure and clinical efficacy of patients in the therapy group were significantly better than those in the control group, and the difference was statistically significant (*P* < 0.05). The results of this study are consistent with the above reports.

When the body's blood coagulation function is abnormal, the blood coagulation function and anticoagulant function in the body will be out of balance (12). The coagulation indexes (TT, PT, Fib, APTT) selected in this study are commonly used indexes for clinical coagulation function tests. TT can indicate the abnormal state of the endogenous coagulation system, PT can indicate the abnormal state of the exogenous coagulation system, Fib can show whether its content in human plasma is at a normal level, and APTT can show the abnormal degree of thrombin activity. It was shown that the blood of patients with pregnancy-induced hypertension was in a hypercoagulable state, with lower TT, PT and APTT levels and higher Fib levels than those of normal pregnant women. The results of this study showed that after therapy, serum TT, PT and APTT levels were higher in the treatment group than in the control group, and Fib levels were lower than in the control group. It was suggested that the combination of nifedipine tablets and magnesium sulfate was effective in improving hypercoagulability in patients with pregnancy-induced hypertension, and was more effective than magnesium sulfate alone. SONG (13) and others have confirmed that nifedipine combined with magnesium sulfate has a good therapeutic effect on patients with pregnancy-induced hypertension, and the clinical effect is significant.

Studies have found that the occurrence and development of hypertension are closely related to the excessive production of reactive oxygen species leading to oxidative stress or the body's ability to resist internal antioxidants (14). In patients with pregnancy-induced hypertension, the activity and content of antioxidant enzymes are found to be low, such as SOD, on the contrary, a large amount of peroxidation products are generated in the body, such as LPO and MDA. Therefore, it is of great significance to explore the level of oxidative stress in patients. The results of this study showed that the oxidative stress level of the two groups of patients after therapy was significantly improved compared with that before treatment, and the serum LPO and MDA of the therapy group were lower than those of the control group, and the SOD level was higher than that of the control group, suggesting that nifedipine tablets combined with magnesium sulfate. It can improve the oxidative stress response of patients with pregnancy-induced hypertension, enhance the body's antioxidant capacity, and reduce the degree of endothelial damage. Vascular endothelial cell injury is one of the pathologies of pregnancy-induced hypertension. After injury, vascular endothelial cells secrete too much vasoconstrictor factor ET, and less secrete vasodilator factor NO (15). ET-1 is the most active endogenous vasoconstrictor peptide found so far, which can promote the proliferation of vascular smooth muscle cells and contract blood vessels. In normal pregnancy, ET-1 is at a normal level, and its increase will cause systemic arteriole spasm, promote the secretion of aldosterone and angiotensin, increase peripheral vascular resistance, and increase blood pressure. The possible mechanism for the increase of ET-1 is that there are endothelial cytotoxic factors that can cause vascular endothelial cell damage in patients with pregnancy-induced hypertension, release a large amount of ET-1 to promote vasoconstriction, and ischemia and hypoxia further aggravate endothelial cell damage, and then form a vicious circle (16). NO is a very active vasodilator released by vascular endothelial cells. It has the effect of inhibiting platelet adhesion, aggregation and vascular smooth muscle cell growth, and can maintain high blood flow and low blood pressure during healthy pregnancy. Studies have reported that the plasma nitrite level in healthy non-pregnant women is lower than that in healthy pregnant women. The level of NO increases significantly in the first trimester and decreases in the third trimester, and returns to normal after delivery, suggesting a significant increase in NO release during pregnancy (17). The dynamic balance between NO and ET can affect the integrity of endothelial cell function to a large extent, and endothelial cell damage can lead to an imbalance between NO and ET (18, 19). Therefore, the abnormal changes in serum ET-1 and NO levels can reflect abnormal vascular endothelial function. The increase in serum ET-1 level and the decrease in NO concentration are one of the mechanisms of the pathogenesis of pregnancy-induced hypertension. Zhang et al. (20) and other studies have confirmed that nifedipine tablets combined with magnesium sulfate in the treatment of hypertension during pregnancy can increase the effective rate of treatment, improve the patient's hemodynamics, and at the same time regulate the patient's plasma ET-1 and NO levels, and improve the function of vascular endothelial cells, and safe and effective. The results of this study showed that after therapy, ET-1 in two groups were significantly increased, and NO and ET-1/NO was significantly decreased, and ET-1 in the therapy group was higher than that in the control group, and NO and ET-1/NO were lower those in the control group. It was suggested that nifedipine tablets combined with magnesium sulfate can improve the degree of vascular endothelial cell damage in patients with pregnancy-induced hypertension by improving serum ET-1 and NO levels, which is consistent with the above view.

In summary, nifedipine tablets combined with magnesium sulfate in the treatment of pregnancy-induced hypertension can improve the coagulation function of patients, reduce oxidative stress damage, regulate the levels of ET-1 and NO in patients' serum, and improve clinical efficacy.

## Data Availability Statement

The original contributions presented in the study are included in the article/supplementary material, further inquiries can be directed to the corresponding author.

## Ethics Statement

The studies involving human participants were reviewed and approved by the Ethics Committee of the Weifang People's Hospital. The patients/participants provided their written informed consent to participate in this study.

## Author Contributions

XY and QZ made equal contributions, including the design of the study, the evaluation of the results, and the writing of the paper. QZ guided the whole study as the supervisor of the whole study. All authors contributed to the article and approved the submitted version.

## Conflict of Interest

The authors declare that the research was conducted in the absence of any commercial or financial relationships that could be construed as a potential conflict of interest.

## Publisher's Note

All claims expressed in this article are solely those of the authors and do not necessarily represent those of their affiliated organizations, or those of the publisher, the editors and the reviewers. Any product that may be evaluated in this article, or claim that may be made by its manufacturer, is not guaranteed or endorsed by the publisher.
